# Establishment of a complex skin structure via layered co-culture of keratinocytes and fibroblasts derived from induced pluripotent stem cells

**DOI:** 10.1186/s13287-018-0958-2

**Published:** 2018-08-13

**Authors:** Yena Kim, Narae Park, Yeri Alice Rim, Yoojun Nam, Hyerin Jung, Kijun Lee, Ji Hyeon Ju

**Affiliations:** 1 CiSTEM laboratory, Catholic iPSC Research Center, College of Medicine, The Catholic University of Korea, 505 Banpo-dong, Seocho-gu, Seoul, 137-701 Republic of Korea; 2 Division of Rheumatology, Department of Internal Medicine, College of Medicine, Seoul St. Mary’s Hospital, The Catholic University of Korea, 505 Banpo-dong, Seocho-gu, Seoul, 137-701 Republic of Korea

**Keywords:** Cord blood mononuclear cell, Induced pluripotent stem cells, Fibroblast, Keratinocyte, 3D skin organoid

## Abstract

**Background:**

Skin is an organ that plays an important role as a physical barrier and has many other complex functions. Skin mimetics may be useful for studying the pathophysiology of diseases in vitro and for repairing lesions in vivo. Cord blood mononuclear cells (CBMCs) have emerged as a potential cell source for regenerative medicine. Human induced pluripotent stem cells (iPSCs) derived from CBMCs have great potential for allogenic regenerative medicine. Further study is needed on skin differentiation using CBMC-iPSCs.

**Methods:**

Human iPSCs were generated from CBMCs by Sendai virus. CBMC-iPSCs were differentiated to fibroblasts and keratinocytes using embryonic body formation. To generate CBMC-iPSC-derived 3D skin organoid, CBMC-iPSC-derived fibroblasts were added into the insert of a Transwell plate and CBMC-iPSC-derived keratinocytes were seeded onto the fibroblast layer. Transplantation of 3D skin organoid was performed by the tie-over dressing method.

**Results:**

Epidermal and dermal layers were developed using keratinocytes and fibroblasts differentiated from cord blood-derived human iPSCs, respectively. A complex 3D skin organoid was generated by overlaying the epidermal layer onto the dermal layer. A humanized skin model was generated by transplanting this human skin organoid into SCID mice and effectively healed skin lesions.

**Conclusions:**

This study reveals that a human skin organoid generated using CBMC iPSCs is a novel tool for in-vitro and in-vivo dermatologic research.

**Electronic supplementary material:**

The online version of this article (10.1186/s13287-018-0958-2) contains supplementary material, which is available to authorized users.

## Background

Skin, the largest organ, covers the exterior of the body and protects internal organs. It has diverse functions and is involved in protection against pathogens, storage of water, regulation of body temperature, and excretion of body waste [1, 2]. Wounds that extend deep into the dermis heal very poorly (especially the epithelium) due to a lack of keratinocytes [3]. Traditionally, there are two main types of skin transplantation, namely allografts and autografts. An autograft is usually the treatment of choice because there is no risk of rejection. Skin grafting is mainly performed in people with severe skin lesions, such as skin cancer patients and burn victims. However, skin biopsy is a highly invasive procedure if a large area needs to be covered. In patients with extensive burns, the skin graft is meshed and typically stretched 3-fold to cover the large area. Use of a harvested skin graft that is unmeshed and has a thicker dermal layer compromises the healing process [3]. Due to the limited availability of autogenous donor tissue, the primary substitute is usually an allograft [4]. Unfortunately, an allograft is typically rejected by the host’s immune system after about 1 week and is therefore usually only used temporarily until an autograft can be applied [3, 5].

One strategy for overcoming immune rejection is to isolate a proliferative cell source with the same immune identity as the recipient. Human induced pluripotent stem cells (iPSCs) are emerging as a promising alternative in this regard [6–8]. Human iPSCs are generated from somatic cells via transduction of reprogramming factors, such as OCT4, SOX2, Klf4, and c-Myc. Reprogrammed iPSCs overcome the ethical and immunological issues associated with embryonic stem cells (ESCs) and can differentiate into multiple lineages.

Human leukocyte antigen (HLA) is the most polymorphic gene in humans and is related to the major histocompatibility complex, which regulates immune responses and the rejection of foreign organs. Cord blood mononuclear cells (CBMCs) have emerged as a potential cell source for regenerative medicine because HLA typing is mandatory during the CBMC banking process, meaning that research materials can be obtained easily [9, 10]. CBMCs are widely used to convert cord blood banks into iPSC banks, which hold great potential in allogenic regenerative medicine. Generation of cell lines from homozygous HLA-typed iPSCs obtained from CBMC-iPSC banks is a novel and efficient strategy for cell-based therapy [9–11]. CBMC-iPSCs have been differentiated into cardiomyocytes, hepatocytes, and chondrocytes [12–17]. However, the differentiation of CBMC-iPSCs into skin cells has not been reported to our knowledge.

In this study, we used CBMC-iPSCs which were generated and characterized in our previous report [8, 9, 16]. Keratinocytes and fibroblasts are the main cellular components of the epidermis and dermis, respectively. CBMC-iPSCs were differentiated into keratinocytes and fibroblasts, and the differentiated cells were stratified as 3D layers. This study suggests that a human skin organoid generated using CBMC-iPSCs is a novel tool for in-vitro and in-vivo dermatologic research.

## Methods

### Differentiation of CBMC-derived iPSCs into fibroblasts

CBMC-derived iPSCs were generated and characterized as described previously [9]. In our previous study, we generated 13 homozygous HLA CBMC-derived iPSC lines that had highly frequent types of HLA in South Korea. Also, CBMC-derived iPSC lines were characterized with high pluripotency, normal karyotypes, and ability to differentiate into three germ layers. We used CMC-hiPSC-011 cell lines in this study. For fibroblast differentiation, embryonic bodies (EBs) were attached to a Matrigel-coated plate on day 0. Fibroblast differentiation medium 1 (FDM1) was a mixture of Dulbecco’s modified Eagle medium (DMEM) and F12 medium (3:1) supplemented with 5% fetal bovine serum (FBS), 5 μg/ml insulin, 0.18 mM adenine, and 10 ng/ml epidermal growth factor (EGF; R&D, MN, USA). Cells were cultured in this medium from day 0 to day 3 and then in the same medium supplemented with 0.5 nM bone morphogenetic protein 4 (BMP4; R&D) from day 4 to day 6. At day 7, the medium was changed to fibroblast differentiation medium 2 (FDM2), which was a mixture of DMEM and F12 medium (1:1) supplemented with 5% FBS and 1% nonessential amino acids. Cells were passaged onto noncoated and type I collagen-coated dishes (BD Biosciences, NJ, USA) on day 14 and day 21, respectively, in FDM1. Primary fibroblasts were grown in DMEM containing 10% FBS. Differentiation was repeated five times per experiment. For induction of fibrosis, iPSC-Fs were treated with 10 ng/ml TGF-β1 (Peprotech, NJ, USA) for 2 days. Antifibrotic agent Pirfenidone (Sigma-Aldrich, MO, USA) was used to treat iPSC-Fs for 24 h. Pirfenidone was diluted in DMSO at 1 mg/ml for treatment.

### Differentiation of CBMC-derived iPSCs into keratinocytes

Human iPSCs were detached using trypsin–EDTA, resuspended in Aggrewell medium (1 × 10^4^ cells/drop; STEMCELL Technologies, BC, Canada), and hung on a 100-mm plate lid. The next day, aggregated EBs were transferred to a 100-mm Petri dish and incubated overnight in TeSR-E8 medium (STEMCELL Technologies) at 37 °C in 5% CO_2_. For keratinocyte differentiation, EBs were attached to a plate coated with type IV collagen (Santa Cruz, CA, USA) on day 0. Keratinocyte differentiation medium 1 (KDM1) was a mixture of DMEM and F12 medium (3:1) supplemented with 2% FBS, 0.3 mmol/l l-ascorbic acid, 5 μg/ml insulin, and 24 μg/ml adenine. Cells were cultured in this medium from day 1 to day 7. Keratinocyte differentiation medium 2 (KDM2) was keratinocyte serum-free media supplemented with 0.3 mmol/l l-ascorbic acid, 5 μg/ml insulin, and 10 μg/ml adenine. Cells were cultured in this medium from day 8 to day 11. Keratinocyte differentiation medium 3 (KDM3) was a mixture of defined keratinocyte serum-free media and keratinocyte serum-free medium (1:1). Cells were cultured in this medium from day 12 to day 30. Keratinocyte differentiation media 1 and 2 were supplemented with 1 μg/ml retinoic acid (RA; Sigma-Aldrich), 25 ng/ml BMP4, and 20 ng/ml EGF. Keratinocyte differentiation medium 3 was supplemented with 25 ng/ml BMP4 and 20 ng/ml EGF. Primary keratinocytes were maintained in defined keratinocyte serum-free medium.

### Culture of a 3D iPSC-derived skin organoid (iSO)

Differentiated fibroblasts were resuspended in neutralized type I collagen at a density of 2 × 10^5^ cells/ml. This suspension was added into each insert of a Transwell plate (Life Technologies, CA, USA) and incubated for 5–7 days in fibroblast differentiation medium 1. Thereafter, 1×10^6^ keratinocytes were seeded onto the fibroblast layer in low-calcium epithelial medium (EP1), cultured for 2 days, and then submerged in normal-calcium medium (EP2) for 2 days. Thereafter, medium was only added to the lower chamber of the insert to generate an air-liquid interface (EP3). A 3D skin organoid was generated from primary cells using the same protocol as a control.

### Transplantation of the 3D iSO

All procedures involving animals were performed in accordance with the Laboratory Animals Welfare Act, the Guide for the Care and Use of Laboratory Animals, and the Guidelines and Policies for Rodent Experimentation provided by the Institutional Animal Care and Use Committee of the School of Medicine of The Catholic University of Korea. The study protocol was approved by the Institutional Review Board of The Catholic University of Korea (CUMC-2017-0150-01). Skin measuring about 1 cm × 2 cm was removed from male NOD/SCID mice (6 weeks old; Jackson, Bar Harbor, ME, USA). The human iSO was washed with PBS, placed into the defect site, and sutured using the tie-over dressing method and silk suture. Mice were sacrificed after 2 weeks for histological analysis.

### Polymerase chain reaction

Total mRNA was extracted using Trizol (Life Technologies) and cDNA was synthesized using a RevertAid First Strand cDNA Synthesis Kit (Thermo Fisher Scientific). Quantitative real-time PCR was performed using SYBR Green Mix and was carried out using a LightCycle 480 Instrument II (Roche, Basel, Switzerland). Primer sequences are presented in Table 1.

**Table 1:** Sequence of primers used for quantitative real-time polymerase chain reaction

Target name	Direction	Primer sequence (5′–3′)	Size (base pairs)
OCT4	Forward	ACCCCTGGTGCCGTGAA	190
	Reverse	GGCTGAATACCTTCCCAAATA	
CD44	Forward	AAGGTGGAGCAAACACAACC	151
	Reverse	AGCTTTTTCTTCTGCCCACA	
COL1A1	Forward	CCCCTGGAAAGAATGGAGATG	148
	Reverse	TCCAAACCACTGAAACCTCTG	
COL1A2	Forward	GGATGAGGAGACTGGCAACC	77
	Reverse	TGCCCTCAGCAACAAGTTCA	
COL3A1	Forward	CGCCCTCCTAATGGTCAAGG	161
	Reverse	TTCTGAGGACCAGTAGGGCA	
Vimentin	Forward	GAGAACTTTGCCGTTGAAGC	170
	Reverse	TCCAGCAGCTTCCTGTAGGT	
PAX6	Forward	GTCCATCTTTGCTTGGGAAA	110
	Reverse	TAGCCAGGTTGCGAAGAACT	
SOX1	Forward	CACAACTCGGAGATCAGCAA	133
	Reverse	GGTACTTGTAATCCGGGTGC	
Np63	Forward	GGAAAACAATGCCCAGACTC	294
	Reverse	GTGGAATACGTCCAGGTGGC	
KRT5	Forward	ACCGTTCCTGGGTAACAGAGCCAC	198
	Reverse	GCGGGAGACAGACGGGGTGATG	
KRT14	Forward	GCAGTCATCCAGAGATGTGACC	181
	Reverse	GGGATCTTCCAGTGGGATCT	
GAPDH	Forward	ACCCACTCCTCCACCTTTGA	110
	Reverse	CTGTTGCTGTAGCCAAATTCGT	

### Histological analysis

Skin was fixed in 4% paraformaldehyde at room temperature, and then dehydrated and cleared using graded ethanol and xylene. After paraffin infiltration and embedding, paraffin blocks of the iSO were sectioned at a thickness of 8 μm using a microtome. Slides were dried for 60 min at 60 °C and deparaffinized by two incubations with xylene. Sections were rehydrated using a decreasing sequential ethanol series and rinsed under tap water for 5 min. For hematoxylin and eosin (H&E) staining, sections were incubated in Harris’ hematoxylin solution for 10 min, washed with 1% HCl–ethanol, neutralized in 0.2% ammonia water, and counterstained with eosin for 1 min. For Picrosirius Red (PSR) staining, slides were incubated in PSR solution for 1 h and washed with acetic acid. For Masson’s trichrome staining, slides were refixed in Bouin’s solution overnight at room temperature and incubated in Weigert’s hematoxylin for 10 min, Biebrich scarlet-acid fuchsin for 5 min, and a mixture of phosphotungstic acid, phosphomolybdic acid, and distilled water (1:1:2) for 10 min. Thereafter, slides were directly transferred to 2% aniline blue, incubated for 5 min, washed with 1% acetic acid, and then incubated in an increasing sequential ethanol series. Ethanol was cleared using xylene and slides were mounted using VectaMount permanent mounting medium (Vector Laboratories, Burlingame, CA, USA). Staining was examined underneath a bright-field microscope.

### Immunohistochemical analysis

Slides were dried at 60 °C and deparaffinized by two cycles of xylene. Slides were rehydrated and endogenous peroxidase activity was blocked by 3% hydrogen peroxide. Slides were washed and blocked with phosphate-buffered saline (PBS) with 1% bovine serum albumin. Primary antibodies were diluted in blocking solution and incubated at 4 °C overnight. The next day, slides were washed with PBS containing 0.1% Tween 20. Secondary antibodies were diluted in blocking solution and treated for 10 min at RT. After washing, slides were treated with ABC reagent for 10 min. DAB solution was followed and incubated for 1 min. Slides were washed and counterstained with Mayer’s hematoxylin for 1 min. Slides were dehydrated and cleared. Slides were mounted and staining was confirmed under a bright-field microscope.

### Statistical analysis

Statistical analyses were performed using GraphPad Prism software. The results are shown as mean and standard error of the mean. Error bars represented the standard error of the mean. Differences between groups were examined for statistical significance using Student’s *t* test. The *t* test was applied to analyze nonparametric quantitative datasets, and the one-tailed *p* value was calculated (*p* < 0.05, *p* < 0.01, and *p* < 0.001 indicated statistical significance.)

## Results

### Generation of CBMC-iPSC-derived fibroblasts

**Fig. 1:**
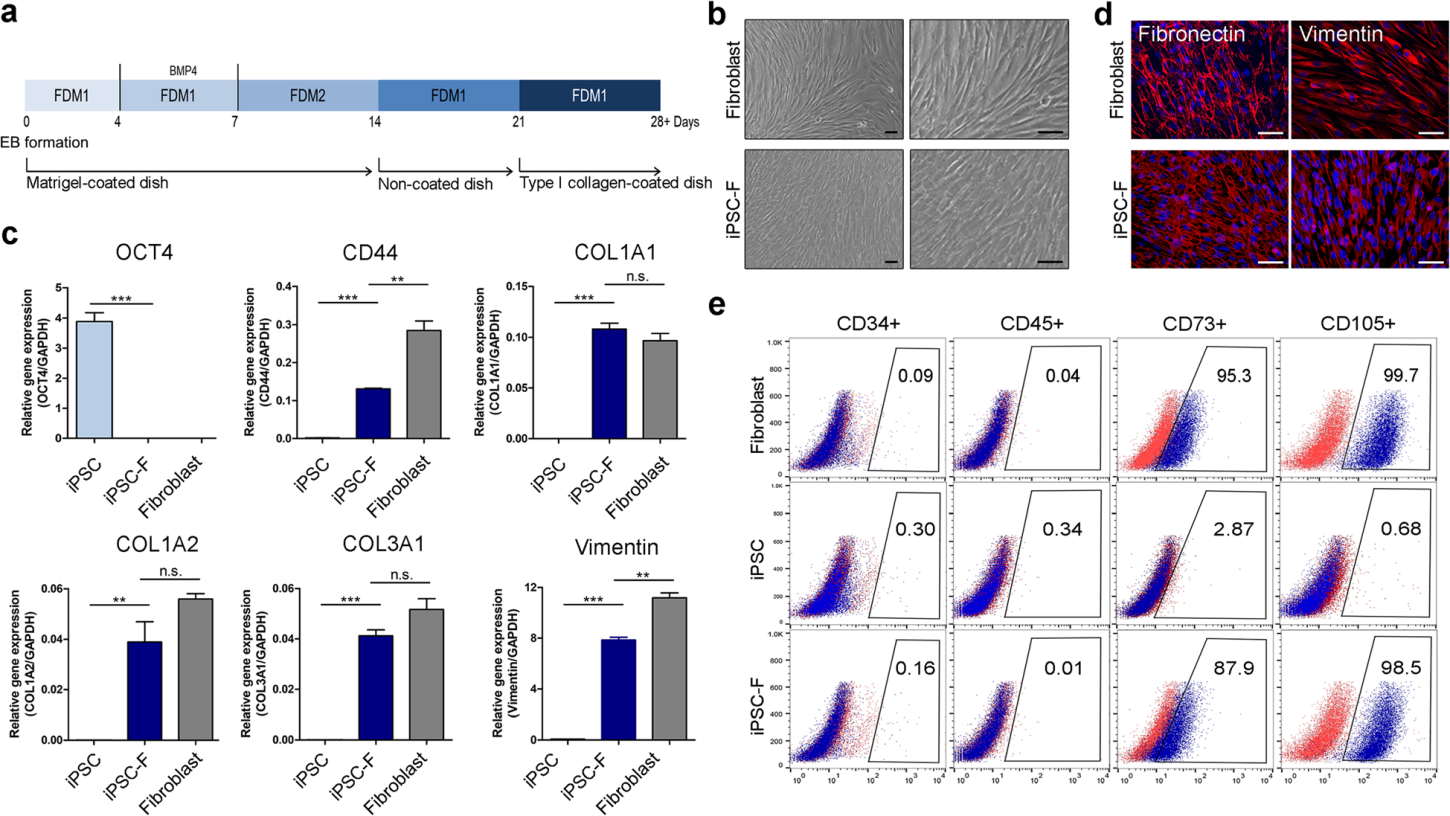
Differentiation of CBMC-iPSC-Fs. **a** Schematic of fibroblast differentiation process. **b** Morphology of iPSC-Fs on day 30. Scale bars, 100 μm. **c** Gene expression of pluripotency marker OCT4 and fibroblast markers CD44, COL1A1, COL1A2, COL3A1, and vimentin. **d** Immunocytochemical analysis of fibroblast markers fibronectin (red) and vimentin (red), together with DAPI staining (blue). Scale bars, 100 μm. **e** Flow cytometric analysis of CD34-positive, CD45-positive, CD73-positive, and CD105-positive cells. Graphs show mean with SEM of five independent samples. **p < 0.01, ***p < 0.001. EB embryoid body, FDM fibroblast differentiation medium, GAPDH glyceraldehyde 3-phosphate dehydrogenase, iPSC induced pluripotent stem cell, iPSC-F iPSC-derived fibroblast, n.s. not significant

To generate CBMC-iPSC-derived fibroblasts (iPSC-Fs), cell outgrowth from EBs was induced and then fibroblasts were differentiated. A scheme of the fibroblast differentiation protocol is shown in Fig. 1a (*n* = 5 CBMC-derived iPSC lines per experiment). EBs were transferred to Matrigel-coated dishes and then differentiated cells were passaged onto noncoated and type I collagen-coated dishes. CBMC-iPSC-Fs had similar morphology to 3T3 cells, an established fibroblast cell line, on day 30 (Fig. 1b). Gene expression of the pluripotency marker OCT4 was lower in iPSC-Fs than in iPSCs. CBMC-iPSC-Fs expressed various markers of fibroblasts, including CD44, COL1A1, COL1A2, COL3A1, and vimentin (Fig. 1c). Fibronectin and vimentin are well-known markers used to characterize fibroblasts. Immunocytochemistry confirmed that expression of these proteins was increased in iPSC-Fs (Fig. 1d). Expression of iPSC marker SOX2 was downregulated in iPSC-Fs (see Additional file 1a). Flow cytometric analysis showed that hematopoietic markers (i.e., CD34 and CD45) were less expressed in iPSCs, and fibroblast markers (i.e., CD73 and CD105) were also less expressed in iPSCs. After differentiation, iPSC-Fs showed low expression of CD34 and CD45. However, expression of CD73 and CD105 was increased in iPSC-Fs and was similar to primary fibroblasts. Through these results, we confirmed that iPSCs were able to differentiate into fibroblasts.

### Treatment of iPSC-Fs with profibrotic and antifibrotic agents

**Fig. 2:**
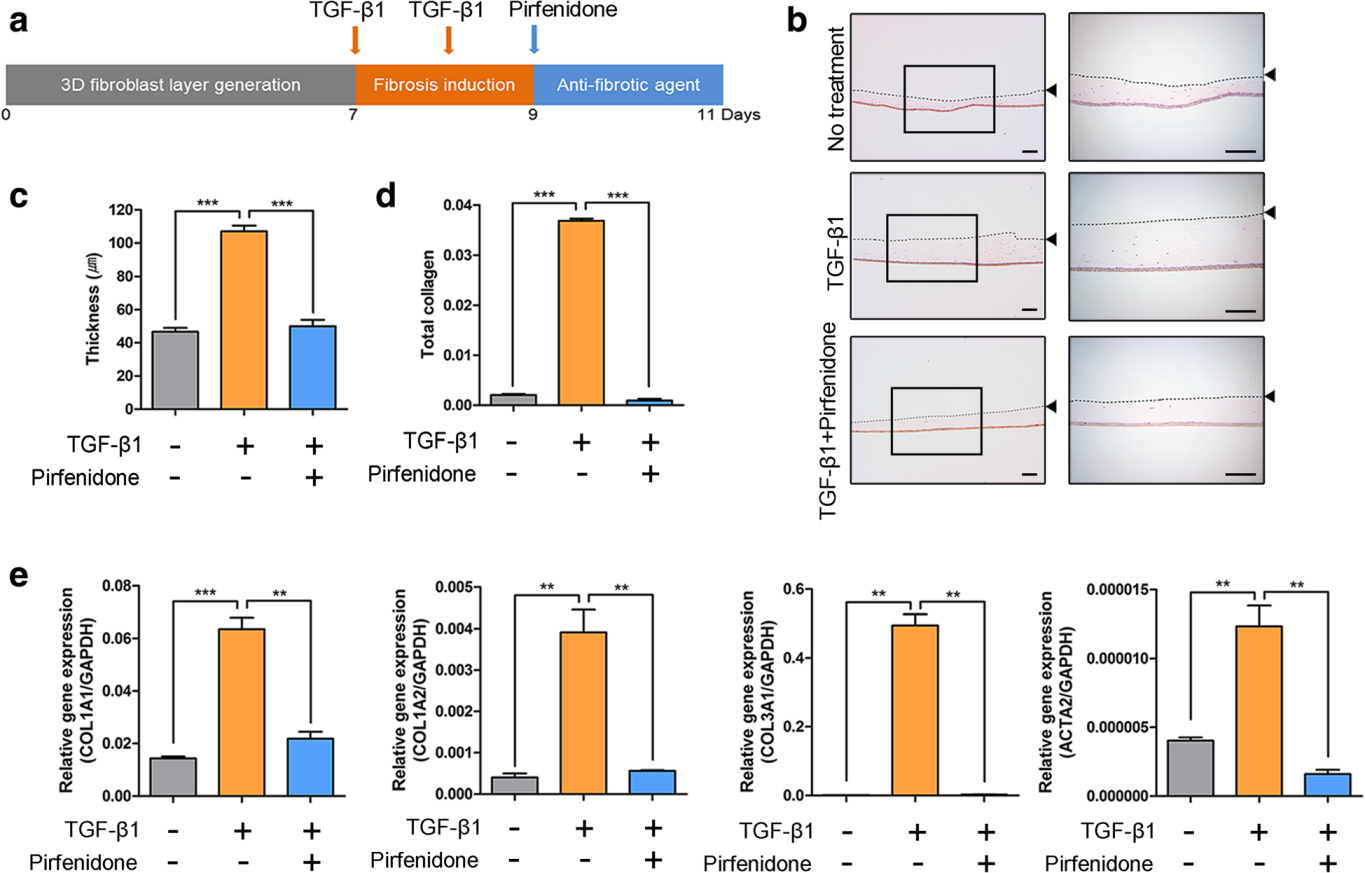
Application of drug test platform by CBMC-iPSC-Fs. **a** Schematic for treatment of iPSC-Fs with TGF-β1 and pirfenidone. **b** Changes in thickness of 3D iPSC-F layer following TGF-β1 and pirfenidone treatment. Dotted lines and arrowheads indicate upper surface of 3D iPSC-F layer. Scale bars, 200 μm. **c** Effects of pirfenidone and TGF-β1 on skin thickness. **d** Quantification of total collagen content using hydroxyproline assay. **e** Gene expression of fibrotic markers COL1A1, COL1A2 COL3A1, and ACTA2 upon treatment with pirfenidone and TGF-β1. Graphs show mean with SEM of five independent samples. **p < 0.01, ***p < 0.001. GAPDH glyceraldehyde 3-phosphate dehydrogenase, TGF transforming growth factor

3D culture of iPSC-Fs was performed using type I collagen. Transforming growth factor (TGF)-β1 is a major cytokine with profibrotic effects. We investigated whether iPSC-Fs were sensitive to TGF-β1 (Fig. 2a; *n* = 5 layers of 3D iPSC-Fs per experiment). Treatment with TGF-β1 activated iPSC-Fs and increased their proliferation rate and extracellular matrix (ECM) production. In addition, TGF-β1 treatment increased the thickness of the 3D iPSC-F layer. Pirfenidone, a drug used to treat idiopathic pulmonary fibrosis, elicits an antifibrotic effect. Treatment with pirfenidone attenuated the increase in the thickness of the 3D iPSC-F layer induced by TGF-β1 (Fig. 2b, c). Hydroxyproline is a component of collagen and was used to quantify the level of total collagen. The total collagen level was increased by TGF-β1 treatment and this effect was attenuated by pirfenidone treatment (Fig. 2d). Gene expression of the fibrosis markers COL1A1, COL1A2, COL3A1, and ACTA2 was increased by TGF-β1 treatment and this effect was attenuated by pirfenidone treatment (Fig. 2e). In summary, the 3D CBMC-iPSC-F layer responded to TGF-β1 treatment, while pirfenidone treatment inhibited fibrosis, as confirmed by the decrease in the layer thickness. These results suggest that iPSC-Fs are applicable for drug screening.

### Generation of CBMC-iPSC-derived keratinocytes

**Fig. 3:**
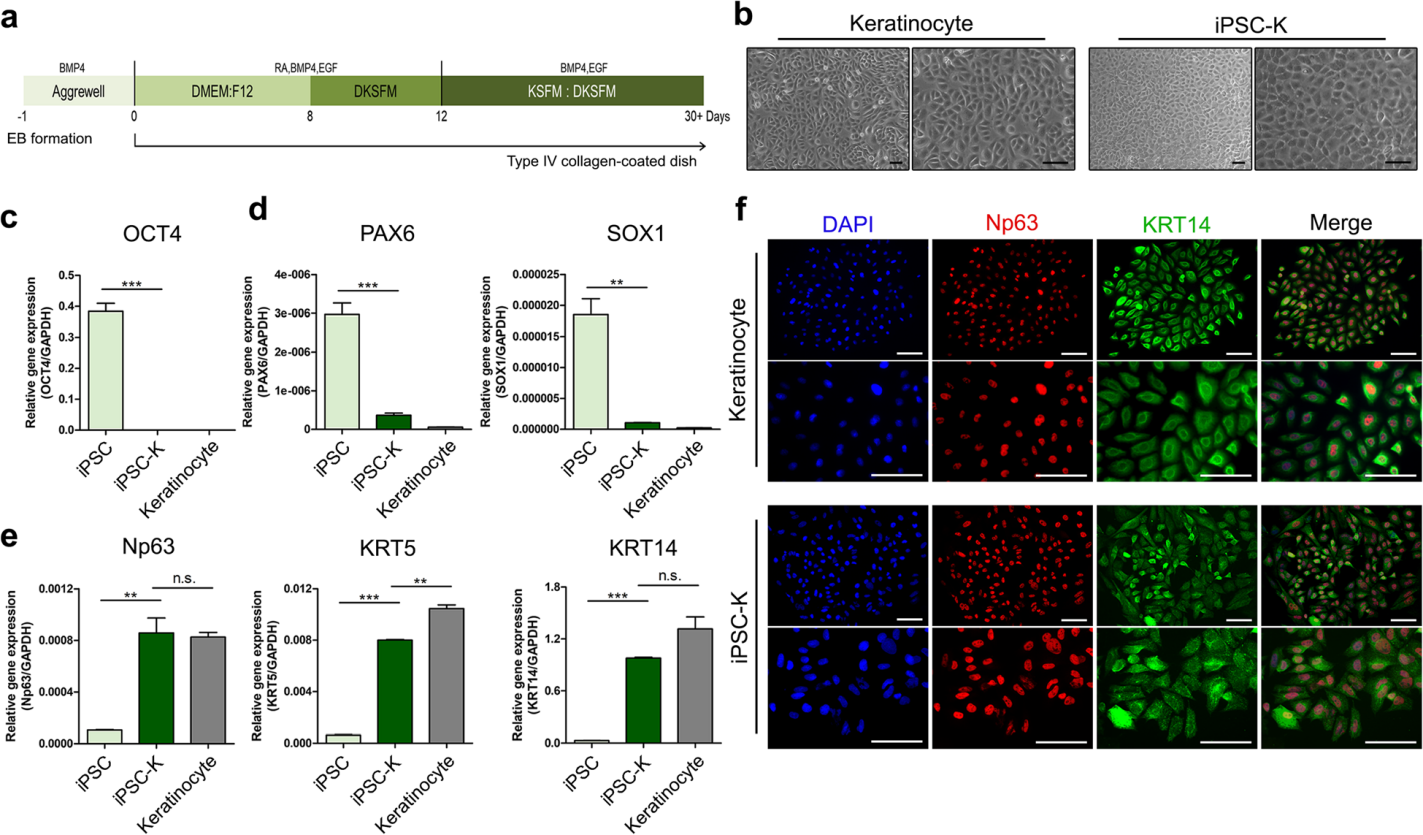
Differentiation of iPSC-Ks and generation of iSO. **a** Schematic of iPSC-K differentiation process. **b** Morphology of iPSC-Ks on day 14. Scale bars, 100 μm. **c–e** Gene expression of (**c**) iPSC marker (OCT4), (**d**) neuroectoderm markers (PAX6 and SOX1), and (**e**) keratinocyte markers (Np63, KRT5, and KRT14). **f** Immunocytochemical analysis of Np63 (red) and KRT14 (green), together with DAPI staining (blue). Scale bars, 100 μm. Graphs show mean with SEM of five independent samples. **p < 0.01, ***p < 0.001. BMP4 bone morphogenetic protein 4, DAPI 4′,6-diamidino-2-phenylindole, DKSFM defined keratinocyte serum free medium, DMEM Dulbecco’s modified Eagle medium, EGF epidermal growth factor, GAPDH glyceraldehyde 3-phosphate dehydrogenase, iPSC induced pluripotent stem cell, iPSC-K iPSC-derived keratinocyte, KSFM keratinocyte serum free medium, n.s. not significant, RA retinoic acid

To apply iPSC-Fs in a more realistic setting, we also differentiated CBMC-derived iPSCs into keratinocytes (iPSC-Ks), another representative skin cell type. Keratinocyte differentiation was performed via induction and maturation of keratinocytes. A scheme of the keratinocyte differentiation protocol is shown in Fig. 3a (*n* = 5 iPSC lines per experiment). CBMC-derived iPSCs were treated with 1 μg/ml RA, 25 ng/ml BMP4, and 20 ng/ml EGF to generate keratinocytes. We generated EBs using the hanging drop method to ensure uniform and well-controlled differentiation. EBs were transferred to type IV collagen-coated dishes. iPSC-Ks expanded from EBs had a primary keratinocyte-like cobblestone morphology when cultured on type IV collagen-coated dishes (Fig. 3b). Expression of the pluripotency marker OCT4 and the neuroectoderm markers PAX6 and SOX1 was decreased in iPSC-Ks (Fig. 3c, d). These results indicate that iPSC-Ks lacked characteristics of iPSCs and did not differentiate along the neuroectoderm lineage. Gene expression of the keratinocyte markers Np63, KRT5, and KRT14 was increased in iPSC-Ks at day 21 (Fig. 3e). Downregulated expression of OCT4 was confirmed in iPSC-Ks by immunohistochemical staining (see Additional file 1b). Expression of Np63 and KRT14, however, was upregulated in iPSC-Ks (Fig. 3f) and was similar to that in primary keratinocytes. These results demonstrated that iPSC-Ks were similar to primary keratinocytes in terms of their gene expression and morphology.

### Generation of an in-vitro 3D iSO and its transplantation into mice

**Fig. 4:**
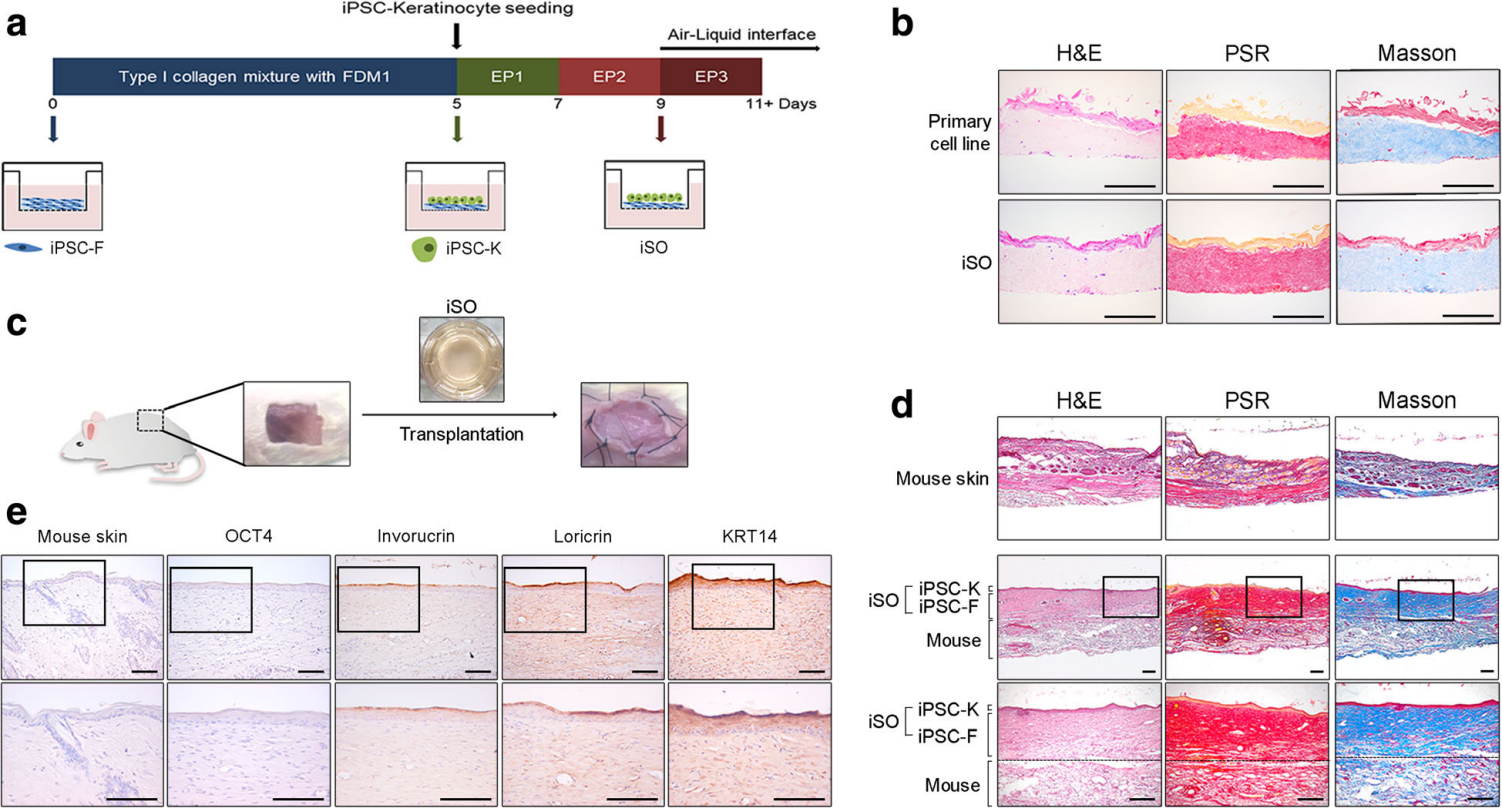
Generation of CBMC-iPSC-derived skin organoid and humanized mice model. **a** Schematic of iSO generation process. **b** Histological analysis of iSO in vitro. Scale bars, 200 μm. **c** Transplantation of iSO into model mice. **d** Histological analysis of mouse skin and transplanted iSO. **e** Immunohistochemical analysis of mouse skin and transplanted iSO with OCT4, involucrin, loricrin, and KRT14. Scale bars, 200 μm. Graphs show mean with SEM of five independent samples. *p < 0.05, **p < 0.01, ***p < 0.001. FDM fibroblast differentiation medium, EP epithelial medium, H&E hematoxylin and eosin, iPSC induced pluripotent stem cell, iPSC-F iPSC-derived fibroblast, iPSC-K iPSC-derived keratinocyte, iSO induced pluripotent stem cell derived skin organoid, PSR Picrosirius Red

Keratinocytes and fibroblasts are the major cell types in the epidermal and dermal layers of skin, respectively. We produced a 3D iSO on a Transwell plate using iPSC-Ks and iPSC-Fs. iPSC-Fs were stratified in type I collagen and overlaid with iPSC-Ks. At 4 days after seeding iPSC-Ks (day 9), the medium in the upper chamber was removed and high-calcium medium was added only into the lower chamber to generate an air–liquid interface (Fig. 4a). This induced keratinocyte maturation and the formation of stratified layers. The iSO was cultured in an upward-facing orientation during formation of the 3D layered structure. The iSO increased in thickness during the formation of the 3D layered structure. In addition, a 3D skin organoid was derived from primary cell lines via the same method (*n* = 5 3D skin samples per experiment). The iSO was analogous to this primary cell line-derived 3D skin organoid (Fig. 4b). We transplanted the iSO into NOD/SCID mice. A skin defect (approximately 1 cm × 2 cm) was surgically induced and the iSO was transplanted using the tie-over dressing method (Fig. 4c). The iSO was well transplanted into the defect lesion (*n* = 5 mice per experiment; Fig. 4d). Furthermore, we confirmed that the transplanted 3D skin graft functionally and terminally differentiated into a skin-like structure in vivo by immunohistochemical staining (Fig. 4e). Pluripotency marker of OCT4 was not expressed in transplanted iSO. Epidermal differentiation markers of involucrin and loricrin, however, were upregulated. These results suggest that the iSO was able to differentiate similarly to the 3D skin organoid generated from primary cells. We also confirmed that the iSO was functionally differentiated and efficiently transplanted into mice and healed defects when transplanted into mice.

## Discussion

The generation of personalized human iPSCs has opened up new possibilities for personalized regenerative medicine. Patient-derived personalized iPSCs can overcome the limitations of primary cell sources and the problem of immune rejection. However, the production of such cells is time-consuming, expensive, laborious, and therefore not economically viable. HLA-homozygous iPSCs have been suggested as an alternative to solve this problem. Such cells are thought to be economically valuable because a small number of these cell lines can be applied to a large number of patients.

HLA-typed CBMCs stored in national and private banks worldwide were recently screened to generate HLA-homozygous CBMC-derived iPSCs. We previously sorted HLA-A, HLA-B, and HLA-DRB1 homozygous cell lines that can cover the highest percentage of the South Korean population using the database of the Catholic Hematopoietic Stem Cell Bank of Korea and produced several iPSC lines from CBMCs [9]. Fibroblast-derived iPSCs and ESCs have been differentiated into skin cells. However, skin cells have not been differentiated from CBMC-derived iPSCs to our knowledge.

Protocols for differentiating iPSCs into fibroblasts and keratinocytes were usually performed via EB generation [18, 19]. We attempted to generate EBs in a well-controlled and optimized manner using the hanging drop method, which promotes the generation of uniform EBs [20]. EBs were transferred to Matrigel-coated plates to induce the formation of outgrowth cells, and then fibroblast differentiation was induced via serial passage on noncoated and type I collagen-coated dishes [21–23]. Serial culture on Matrigel-coated, noncoated, and type I collagen-coated plastic is reported to induce and specify fibroblast differentiation. Egles et al. [24] showed that the proliferation rate of fibroblasts increases in the presence of type I collagen. Fibroblasts secrete ECM proteins that are related to cell migration and adhesion [25]. Among the ECM proteins, collagens are the major abundant component secreted by the fibroblasts [26, 27]. CD44 is the surface marker of fibroblasts that plays a role of adhesion [28, 29]. In iPSC-derived fibroblasts, the expression of CD44 was increased compared with iPSCs. Also, the expression of the ECM proteins was upregulated in iPSC-Fs (Fig. 1c). These results mean that gene expression patterns were converted from iPSCs to iPSC-Fs.

In the current study, gene expression of fibroblast markers was increased in iPSC-Fs, whereas that of the pluripotency marker OCT4 was significantly decreased (Fig. 1c, Additional file 1a). Expression of the ECM proteins fibronectin and vimentin was similar in iPSC-Fs and primary fibroblasts (Fig. 1d). Flow cytometric analysis showed that the expression of hematopoietic markers (e.g., CD34 and CD45) was downregulated in iPSCs. Expression of fibroblast markers (e.g., CD73 and CD105) was also downregulated in iPSCs. After differentiation, expression of CD34 and CD45 was also still absent in iPSC-Fs. However, expression of CD73 and CD105 was increased in iPSC-Fs and the levels were similar to that in primary fibroblasts. These results confirmed that iPSCs were able to differentiate into fibroblasts.

To model fibrosis in vitro, iPSC-Fs were treated with TGF-β1, a major profibrotic agent [30]. TGF-β1 treatment increased the proliferation rate and ECM production of iPSC-Fs. By measuring the level of hydroxyproline, a major component of collagen, we confirmed that the total collagen content was increased in TGF-treated iPSC-Fs (Fig. 2d). We also investigated the gene expression levels of fibrosis markers (Fig. 2e). To determine whether iPSC-F layers are applicable for drug screening, we cultured TGF-β1-treated iPSC-Fs in the presence of pirfenidone [31]. This antifibrotic agent has been evaluated in three multinational phase 3 clinical trials involving patients with idiopathic pulmonary fibrosis, which is thought to be related to TGF signaling [32, 33]. Pirfenidone attenuated the increases in the thickness and total collagen content of TGF-β1-treated iPSC-Fs. We confirmed that 3D layers of iPSC-Fs mimic the fibrotic environment and can be used as an in-vitro model to confirm the functions of antifibrotic drugs. A protocol to expand iPSC-Fs on a larger scale may be needed for future use of these cells as a drug-screening platform.

Human skin is mostly composed of dermis and epidermis. To better mimic human skin, we also differentiated CBMC-derived iPSCs into keratinocytes. EBs were attached to plates coated with type IV collagen, a major component of the basement membrane that selectively induces the formation of keratinocytes [34]. Keratinocyte-like cells rapidly attached to type IV collagen. RA and BMP4 are regulators of keratinocyte proliferation and differentiation that induce Np63 during early ectoderm fate and disrupt neural differentiation [35–38]. Treatment with RA and BMP4 induced expression of the progenitor keratinocyte marker Np63 and the mature keratinocyte marker KRT14. These differentiated cells had a cobblestone-like morphology similar to that of primary keratinocytes (Fig. 3b). Gene expression levels of pluripotency markers and neuroectoderm markers were significantly decreased (Fig. 3c–e), while gene expression of keratinocyte markers (i.e., Np63, KRT5, and KRT14) was increased in iPSC-Ks. Expression of Np63 and KRT14 were similar in iPSC-Ks to that in primary keratinocytes (Fig. 3f). These results confirmed that iPSCs were differentiated into keratinocytes and iPSC-Ks exhibited similar expression to that of primary keratinocytes.

A human iSO produced using iPSC-Ks and iPSC-Fs had similar characteristics as a skin organoid generated using primary cell lines (Fig. 4b). Increased production of ECM proteins in the fibroblast region of the iSO was confirmed by various types of staining. On the other hand, iPSC-Ks were organized into stratified layers in the high-calcium and air–liquid interface culture system. A high concentration of calcium is related to keratinocyte differentiation in vivo and in vitro, while the air–liquid interface is thought to develop and maintain multilayered strata [39–43]. We used this protocol to mimic real skin. Staining demonstrated the formation of stratified keratinocytes, which had a similar morphology to real skin. Next, we confirmed the wound-healing ability of the iSO by implanting it into a mouse model using the tie-over dressing method. After 2 weeks, the excised skin graft had a similar morphology to real skin (Fig. 4d). Involucrin and loricrin are epidermal differentiation markers. Involucrin is a marker of differentiated keratinocyte and an essential part of the skin barrier property [44]. Also, loricirin is the main component of the stratum corneum found in terminally differentiated epidermal cells that are expressed in keratinized epithelial cells [45, 46]. This expression suggests that the cells might be terminally differentiated keratinocytes [47–49]. We performed immunohistochemical staining to confirm the expression of these proteins. Immunohistochemical staining results showed that OCT4 was downregulated, while involucrin and loricrin were upregulated along with KRT14 after skin graft to mice (Fig. 4e). These results showed that the iSO was efficiently transplanted in SCID mice skin, and we were able to estimate the regenerated skin structure and the terminally differentiated state of the graft into epidermis. Itoh et al. [19, 50] developed a 3D skin graft using keratinocytes and fibroblasts generated from fibroblast-derived iPSCs. The morphology of our iSO was similar to that of this previously reported artificial skin graft. In addition, our study demonstrated the reconstitution of normal skin on the back of SCID mice using a combination of iPSC-Fs and iPSC-Ks. Tumor formation was not observed. However, the implanted iSO did not show any morphological patterns of hair follicles or glands, similar to previous reports [50]. Lee et al. [51] recently generated a skin organoid that can spontaneously produce hair follicles after inducing self-assembly of epidermis and dermis using mouse iPSCs. In general, most of the previous transplantation experiments were done using single fibroblast and keratinocyte cells in a silicon bubble chamber [52–55]. This system is easy to transplant, but it requires more time for transplantation efficiency. The 3D skin organoid does not require much time to confirm the transplantation efficiency. Also, the 3D skin organoid is more similar to human skin compared to a single cell system. In our 3D skin model of iPSC-derived differentiated cells, we did not use plastic or silicon chambers. When we transplanted the iSO for a long time using our model, mice skin covered many parts of the human iSO. Therefore, to observe the healing ability of the iSO for a longer duration, an optimized in-vivo transplantation method might be useful to further analyze its fate. Also, an optimized protocol to generate an iSO that is more similar to human skin might be helpful for future use.

## Conclusions

We confirmed that CBMC-derived iPSCs can differentiate into keratinocytes and fibroblasts, the main cellular components of human skin. iPSC-Fs responded to a profibrotic agent and confirmed the effects of an antifibrotic drug. An iSO was formed from iPSC-Fs and iPSC-Ks, and implanted into SCID mice. The implanted iSO was successfully maintained for 2 weeks and had a similar morphology to real skin. Our study indicates that CBMC-derived iPSCs are a potential cell source for dermatologic regenerative medicine and can be used for skin-related drug screening. HLA-homozygous iSOs might be a new candidate for allogenic skin grafting. Clinicians only need to ensure that the HLA type of the transplanted cells matches that of the patient. Consequently, iSOs might be immediately applicable for skin grafting and increase the feasibility of skin transplantation in the near future.

## Additional file

Additional file 1: Immunofluorescence analysis of iPSC-Fs and iPSC-Ks with pluripotency markers (SOX2 and OCT4). Scale bars, 100 μm. (TIF 4501 kb)

## Abbreviations

BMP4: Bone morphogenetic protein 4
CBMC: Cord blood mononuclear cell
DMEM: Dulbecco’s modified Eagle medium
EB: Embryonic body
ECM: Extracellular matrix
EGF: Epidermal growth factor
ESC: Embryonic stem cell
FBS: Fetal bovine serum
H&E: Hematoxylin and eosin
HLA: Human leukocyte antigen
iPSC: Induced pluripotent stem cell
iPSC-F: iPSC-derived fibroblast
iPSC-K: iPSC-derived keratinocyte
iSO: Induced pluripotent stem cell-derived skin organoid
PSR: Picrosirius Red
RA: Retinoic acid
TGF: Transforming growth factor
