# Supplementary figures and images for: Establishment of a complex skin structure via layered co-culture of keratinocytes and fibroblasts derived from induced pluripotent stem cells
Source: Stem Cell Res Ther. 2018 Aug 13;9:217. doi: 10.1186/s13287-018-0958-2 (PMC6090613; doi:10.1186/s13287-018-0958-2)

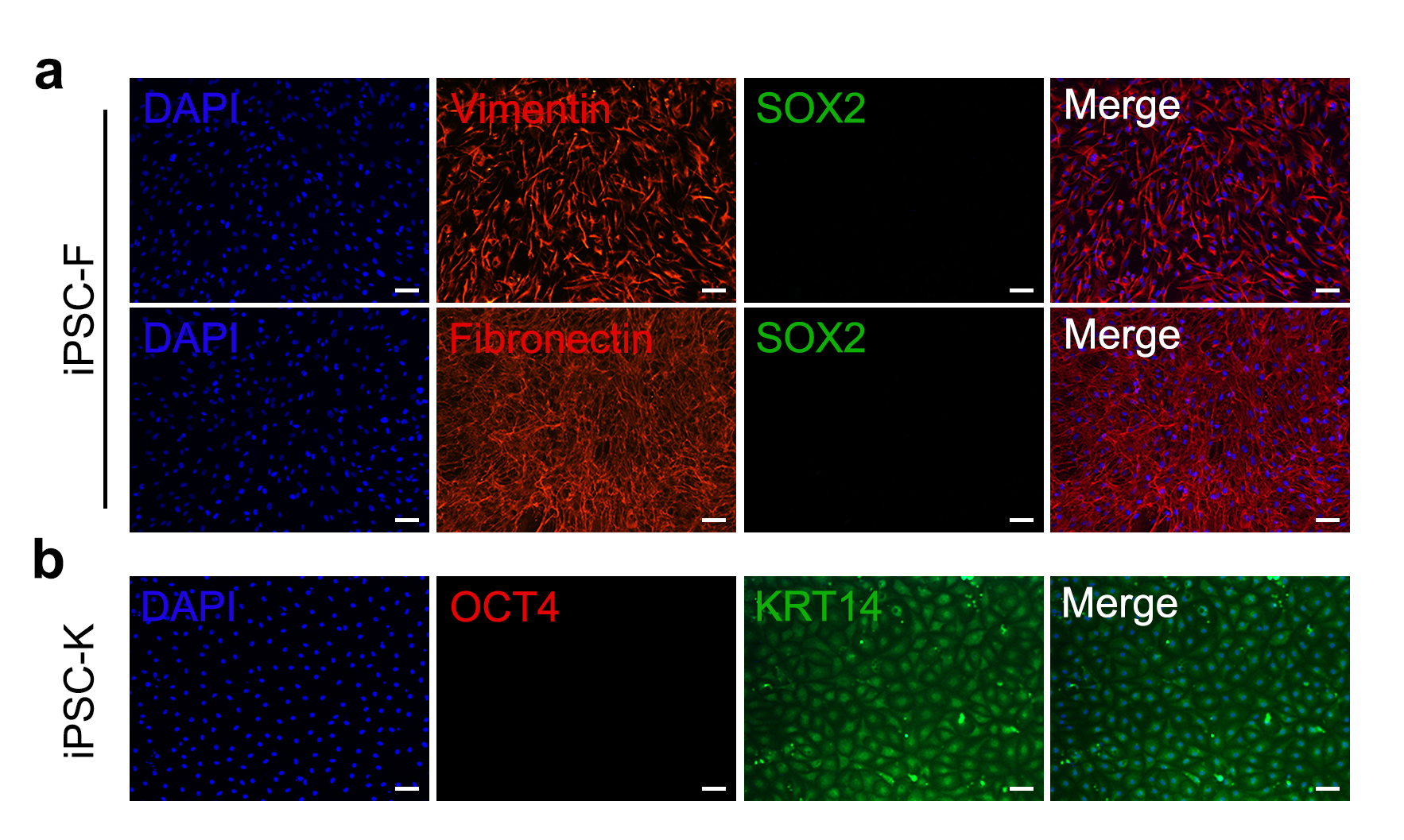

Supplement: Supplementary file 1 — Immunofluorescence analysis of iPSC-Fs and iPSC-Ks with pluripotency markers (SOX2 and OCT4). Scale bars, 100 μm. (TIF 4501 kb) [file 13287_2018_958_MOESM1_ESM.tif]
